# Prelimbic cortex is involved in the regulation of morphine-induced conditioned place preference in both resistant and sensitive mice

**DOI:** 10.1038/s41598-025-87084-7

**Published:** 2025-02-15

**Authors:** Rui Zheng, Yuanyuan Chen, Jin Zhang, Qianglin Liu, Yanyan Zheng, Zhouguang Wang

**Affiliations:** 1 Wenzhou Third Clinical Institute Affiliated to Wenzhou Medical University, Wenzhou People’s Hospital, Wenzhou, 325200 Zhejiang China; 2 Oujiang Laboratory (Zhejiang Lab for Regenerative Medicine, Vision and Brain Health), School of Pharmaceutical Science, Wenzhou Medical University, Wenzhou, 325000 Zhejiang China; 3 Shenzhen Graduate School, Peking University, Peking University, Beijing, 100191 China; 4 Department of Anatomy and K.K. Leung Brain Research Centre, Fourth Military Medical University, Xi’an, 710032 China; 5 School of Basic Medical Sciences, Xi’an Medical University, Xi’an, 710021 China; 6 Biomarker Technologies Ltd, Beijing, 101300 China

**Keywords:** Compulsive, Addiction, Morphine, Prelimbic cortex, RNA-seq, Neuroscience, Psychology

## Abstract

A primary behavioral pathology in drug addiction is the overpowering motivational strength and decreased ability to control the desire to obtain drugs, which shows some variation between different individuals. Here, using a morphine-induced conditioned place preference (CPP) model with footshock, we found that mice exhibited significant individual differences in morphine-induced addiction. Despite the consequences of footshock, a small percentage of mice (24%) still showed stable morphine preference, demonstrating resistant to punishment. The majority of mice (76%) were relatively sensitive to punishment and showed termination of morphine preference. As a region of advanced cognitive function in the mammalian brain, the medial prefrontal cortex (mPFC) is involved in regulating drug-induced addictive behaviors. We found that activating the pyramidal neurons in the prelimbic cortex (PrL) could effectively reverse morphine-induced CPP in resistant mice, and inhibiting pyramidal neurons in the PrL could promote morphine-induced CPP in sensitive mice. To further explore the differences between resistant and sensitive mice, we analyzed the differences in gene expression in their PrL regions through RNA-seq analysis. The results showed that compared to sensitive mice, the significantly downregulated differentially expressed genes (DEGs), such as *Panx2*,* Tcf7l2*,* Htr2c*,* Htr5a*,* Orai3*,* Slc24a4* and *Cacnb2*, in resistant mice were mainly involved in synaptic formation and neurodevelopment. We speculated that there may be defects in the neuronal system of resistant mice, and caused they are more prone to morphine-induced CPP. These findings are likely to contribute to research in gene therapy, and they may also serve as potential therapeutic targets for drug addiction.

**Supplementary Information:**

The online version contains supplementary material available at 10.1038/s41598-025-87084-7.

## Introduction

Addiction is a significant global public health and social issue, typically starting from recreational or voluntary drug use and progressing to compulsive drug-seeking behavior^1,2^. As a chronic relapsing brain disorder, its most core and challenging issue is aberrant learning and memory. Due to the long-lasting associations are formed between psychotropic drug’s effects and the context where they were experienced, so an individual would be driven to compulsively seek and take the drug when exposed to the environment where the drug was previously used^3–5^. Through certain compulsory withdrawal methods, the majority of drug users can become aware of the destructive impact of drug use on their personal and societal well-being, thereby further eliminating drug addiction-related behaviors^2,6^. It is noteworthy that epidemiological studies have found that only a small proportion of drug users lose control over drug use, meeting the diagnostic criteria for addiction^2,7^. These findings suggest that individual differences can influence the progression of drug addiction.

A primary behavioral pathology in drug addiction is the overpowering motivational strength and decreased ability to control the desire to obtain drugs^1,8,9^, which shows some variation between different individuals. For example, the majority of rodents would cease drug-seeking behavior when faced with harmful footshock as punishment in cocaine self-administration, and demonstrating sensitivity to punishment^10,11^. However, about 30% of rodents persisted in cocaine self-administration despite the footshock, showing resistant to punishment, mirroring the clinical situation of compulsive drug use in humans^11^. This suggests that sensitive mice are able to exhibit good self-control and stay away from addictive drugs, while resistant mice may lack self-control. Therefore, exploring the neurobiological differences between these two types of mice may help understand drug addiction-related behaviors and be beneficial for targeted treatment of addicted patients.

As a central part of the mammalian brain, the prefrontal cortex (PFC) is involved in regulating drug-induced addiction, including compulsive seeking and reinstatement^8,12,13^. It has been found that drug addicts, including both humans and rodents, exhibit abnormalities in the PFC, particularly in the medial prefrontal cortex (mPFC)^2,14^. Anatomically, mPFC can be further divided into the prelimbic cortex (PrL), infralimbic cortex (IL), and dorsomedial cortex (DP)^15^. Chen, et al.^11^ described a decrease in excitability in the rat PFC following self-administration of cocaine. Rats with decreased PFC activity also exhibited stronger compulsive drug-seeking behavior, as reflected in their response to drug punishment^11^. Additionally, optogenetic manipulation of mPFC neurons can enhance or impair compulsive cocaine seeking behavior in rodents^11^.

PrL, a homologous to Brodmann area 32 of the anterior cingulate cortex (ACC) in humans^16^, is a subregion of the mPFC that plays a crucial role in various cognitive and behavioral functions, particularly related to decision-making, executive control, addiction-related behaviors, and emotional regulation^17–19^. PrL plays a role in regulating cocaine/morphine addiction in rats, inactivation of the PrL significantly attenuated drug-seeking and drug-taking behaviors in cocaine self-administration^20–22^, and electrical stimulation of PrL suppressed morphine-induced CPP in rats^23^. In addition, PrL also plays a role in seeking of natural, typically food-based, rewards^24,25^. PrL inactivation decreases responding to the rewarded lever in a discriminative-stimulus sucrose seeking task^25,26^, and optogenetic activation of PrL parvalbumin GABAergic interneurons (which presumably inhibit PrL output) enhanced reward extinction learning, but did not influence consumption during conditioning or expression^27,28^. Specifically, the PrL is implicated in contexts related to addiction and emotional processing^12,29^. Thus, we hypothesized that neuronal activity in the PrL may be affect the behavior of both resistant and sensitive mice during drug addiction-related behaviors.

To investigate the differences between resistant and sensitive mice, as well as the molecular mechanisms underlying drug addiction-related behaviors. We used a conditioned place preference (CPP) model with footshock to distinguish between sensitive and resistant mice, and investigated the mechanisms underlying addiction-related behaviors in addiction by chemical-genetic approaches manipulation of neuronal activity in the PrL. Additionally, RNA-seq analysis detected a significant downregulation of genes in the PrL of resistant mice, which may affect neuronal development and function. These findings not only contribute to a better understanding of the mechanisms underlying addiction, but also aid in the development of gene therapies and drugs targeting addiction.

## Results

### A subpopulation of mice displayed morphine-induced conditioned place preference

**Fig. 1:**
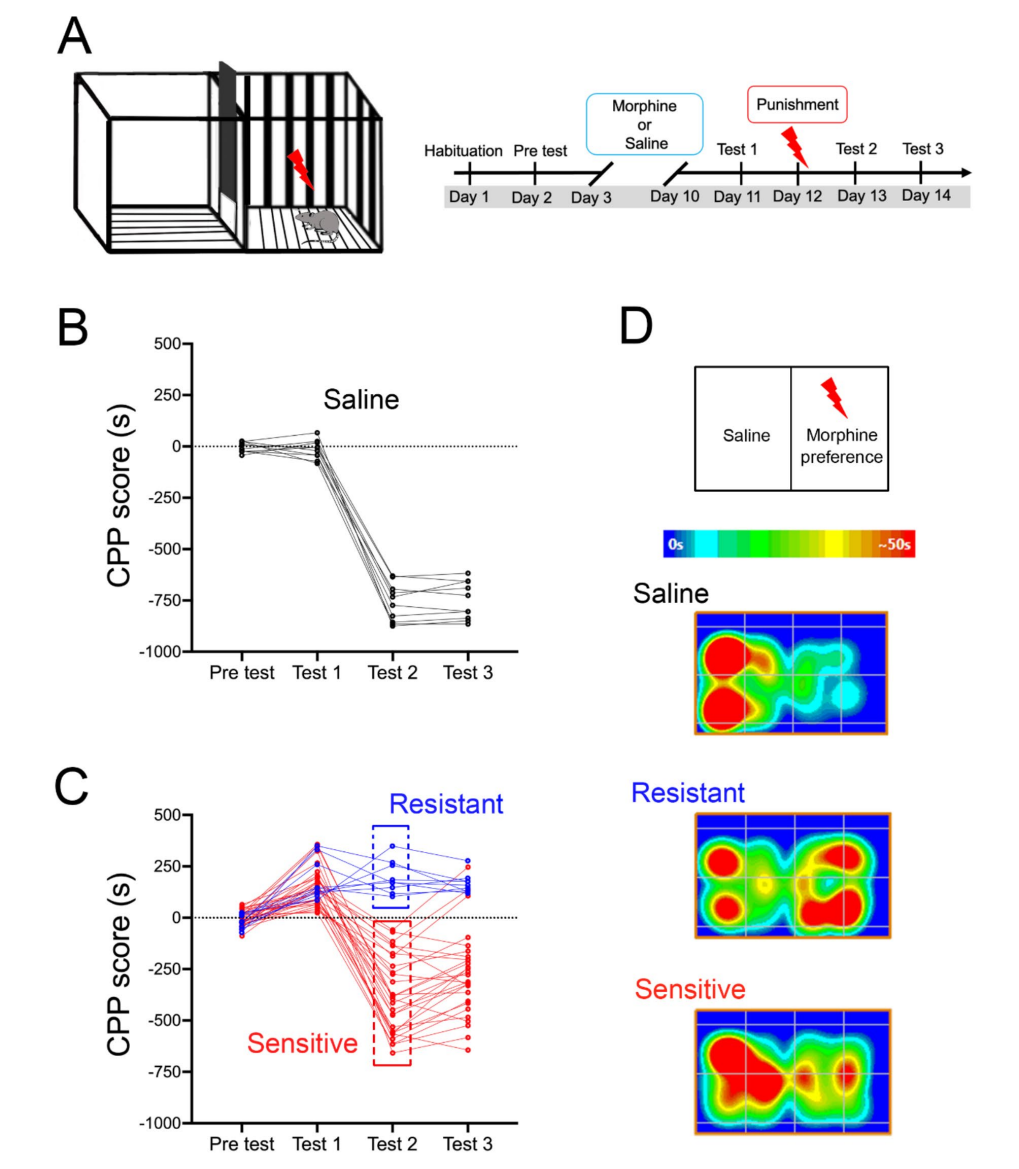
Performance in the CPP task indicates preference of morphine. (**A**) Experimental design of the protocol for CPP. Mice were habituated to 45 min at day 1, and test to choose mice without location preference (Pre test). After habituation, the channel door was closed for drug conditioning, mice were injected with morphine and placed in one side of the chamber for 45 min at day 3, day 5, day 7, day 9, or injected with normal saline and placed on the other side of the chamber for 45 min at day 4, day 6, day 8, day 10. After drug conditioning, we tested for mouse location preferences (Test 1). The next step is the punishment (footshocks) phase, followed by Test 2 and Test 3 to distinguish for punishment-resistant and sensitive mice. The protocol for the control group was similar to that of the CPP experiment, except that saline was used instead of morphine. (**B**) The CPP scores of the control group (Saline) in the CPP experiment of four tests. The mice showed no positional preference and stayed away from the box on the side with the footshock after the punishment. *n* = 10 mice. (**C**) The CPP scores of the test group (morphine) in the CPP experiment of four tests. All mice showed location preference in Test 1, but only a small number of mice continued to show morphine-induced CPP after punishment, showing resistant (blue). Most of the mice stayed away from the box on the side with the shock and showed sensitivity (red). *n* = 37 mice. (**D**) The heatmaps showed the movement tracks of three different groups of mice in Test 2.

To investigate the neurobiological mechanisms underlying addiction-related behavior, we first established a mouse model of addiction induced by morphine. Mice were trained according to the conditioned place preference (CPP) protocol under a chain schedule (Fig. 1A). After 8 days of conditioning training, all mice were entered to Test 1 phase, and the results showed that the mice in the saline group did not show a preference for any location (Fig. 1B), while mice induced with morphine exhibited a clear preference for a specific location (Fig. 1C). Next, the mice began to received punishment trials, unlike the CPP model, the mice received a footshock (0.75 mA, 2 s) whenever they re-entered the morphine-preferred side of the chamber (the chamber which mice stayed for longer time in Tset 1). In subsequent tests (Test 2 and Test 3), we observed two types of mice, shock resistant and shock sensitive mice, and we found that 9 of 37 mice (24%) displaying shock resistant (Fig. 1C, blue), and 26 of 37 mice (76%) displaying shock sensitive (Fig. 1C, red). While mice injected with saline as controls were stayed away from the chamber with footshock, showed that electric shock punishment ensured that mice showed distance from potentially dangerous areas (Fig. 1B and D). Faced with the aversive punishment of footshock, sensitive mice reduced morphine-induced CPP (Fig. 1D, middle), while resistant mice still showed morphine-induced CPP despite the punishment (Fig. 1D, down). Previous studies^11,30^ have observed a similar proportion of shock resistant rat (30%) in cocaine self-administration models, and these resistant rats exhibited stronger baseline impulsivity^31,32^. Although CPP can’t simulate drug-seeking behavior, the morphine-induced CPP could also effectively distinguish resistant and sensitive mice. The above results indicate that individual differences among mice could influence addiction-related behaviors.

### The PrL neuronal activity affects morphine-induced CPP

**Fig. 2:**
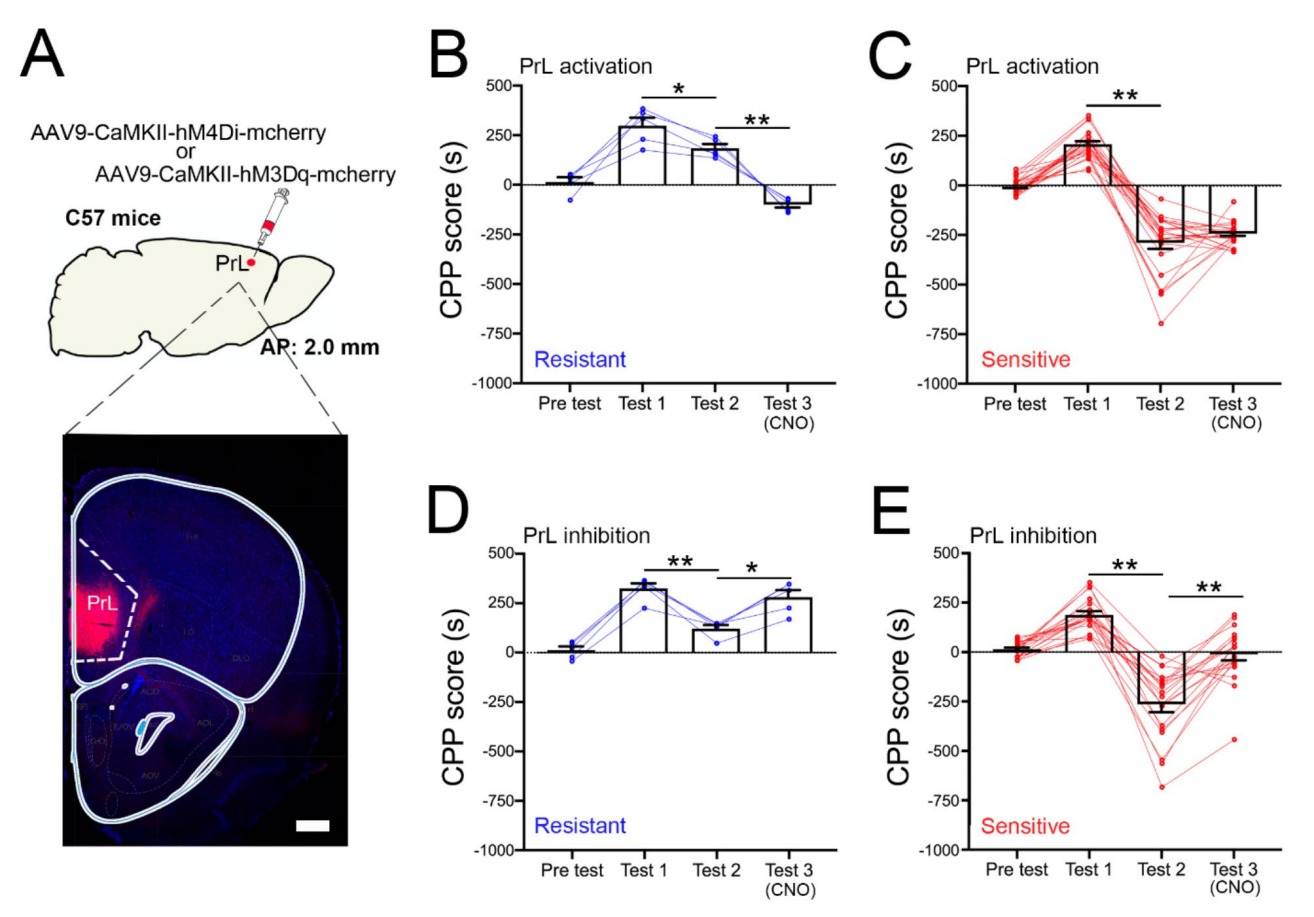
Neuronal activity in PrL affects morphine-induced CPP behavior in resistant and sensitive mice. (**A**) Schematic of the PrL area where the AAV-CaMKII-hM4Di-mcherry or AAV-CaMKII-hM3Dq-mcherry was injected in C57 mice, and representative image of the virus injection site. bar = 1 mm. (**B**-**C**) Using chemogenetics method to activate pyramidal neuron in PrL by injecting AAV-CaMKII-hM3Dq virus and CNO significantly prevented morphine-induced CPP behaviors in resistant mice (**B**), and did not alter morphine-induced CPP behaviors in sensitive mice (**C**). *n* = 5 mice in resistant mice group, *n* = 20 mice in sensitive mice group. (**D**-**E**) Inhibiting PrL pyramidal neuron using CNO and AAV-CaMKII-hM4Di virus led to morphine-induced CPP behaviors in resistant (**D**) and sensitive (**E**) mice. *n* = 5 mice in resistant mice group, *n* = 24 mice in sensitive mice group. * *p* < 0.05, ** *p* < 0.01. Data are presented as means ± SEM.

Clinical studies have found that the decreased function of the medial prefrontal cortex (mPFC) is associated with decline in inhibitory control in addiction-related behaviors^32,33^, and the PrL of the mPFC is involved in a variety of cognitive functions, including decision-making and inhibitory response control^34–36^. To investigate whether morphine-induced CPP is influenced by PrL neural activity, we used a chemogenetic approach to detect the behavioral changes in resistant and sensitive mice. Then AAV-CaMKII-hM3Dq-mCherry was injected into the PrL (Fig. 2A), which has been shown to be able to increase the activity of pyramidal neurons in the PrL^37^. After a 2-week recovery period, mice were trained and tested according to the protocol in Fig. 1A. After punishment and testing, 5 of 25 (20%) mice were identified as resistant (Fig. 2B), and 20 of 25 (80%) the mice were identified as sensitive (Fig. 2C). After a 24-hour recovery period, we activated the pyramidal neurons in the PrL by using clozapine N-oxide (CNO, 3 mg/Kg, i.p.). The results showed a significant decrease in morphine-induced CPP in resistant mice (df = 2, F _(3, 8)_ = 54.24, *P* = 0.0006, Test 1 vs. Test 2, *P* = 0.0382; Test 2 vs. Test 3, *P* = 0.0040; one-way repeated-measure ANOVA (condition) with Bonferroni’s multiple comparisons.) (Fig. 2B), while sensitive mice showed no significant change (df = 2, F _(2, 36)_ = 172.5, *P* < 0.0001, Test 1 vs. Test 2, *P* < 0.0001; Test 2 vs. Test 3, *P* = 0.5753; one-way repeated-measure ANOVA (condition) with Bonferroni’s multiple comparisons.) (Fig. 2C) and still did not exhibit morphine-induced CPP behavior. These results indicating that activation of PrL pyramidal neurons decreased morphine-induced CPP behavior.

Furthermore, to study whether inhibition of PrL pyramidal neurons constituted a sufficient condition for morphine-induced CPP behavior, AAV-CaMKII-hM4Di-mCherry was infused into PrL (Fig. 2A). After a 2-week recovery period, mice were trained and tested according to the protocol in Fig. 1A. After punishment and testing, 5 of 29 (17%) mice were identified as resistant (Fig. 2D), and 24 of 29 (83%) the mice were identified as sensitive (Fig. 2E). After a 24-hour recovery period, we inhibited the pyramidal neurons in the PrL by using CNO. The results showed a significant increase in morphine-induced CPP behavior in resistant mice (df = 2, F _(2, 8)_ = 16.51, *P* = 0.0081, Test 1 vs. Test 2, *P* = 0.01; Test 2 vs. Test 3, *P* = 0.0109; one-way repeated-measure ANOVA (condition) with Bonferroni’s multiple comparisons.) (Fig. 2D), and sensitive mice also exhibited morphine-induced CPP behavior (df = 2, F _(2, 38)_ = 63.02, *P* < 0.0001; Test 1 vs. Test 2, *P* < 0.0001; Test 2 vs. Test 3, *P* < 0.0001; one-way repeated-measure ANOVA (condition) with Bonferroni’s multiple comparisons) (Fig. 2E). These results indicating that inhibitor of PrL pyramidal neurons increased morphine-induced CPP behavior.

### RNA-seq analysis of the differences between resistant and sensitive mice

The activity of PrL neurons affects the addiction-related behaviors of resistant and sensitive mice, but it is still unknown the underlying reasons for the differences between resistant and sensitive mice during the drug addiction process. We hypothesized that phenotypic differences among individual mice may be influenced by neurodevelopment. Therefore, we used RNA-Seq to detect differences in gene expression in the PrL to investigate the differences between resistant and sensitive mice. The results showed that a total of 1378 genes were differentially expressed in the resistant mice compared to the sensitive mice, with 650 genes upregulated and 728 genes downregulated (Table S1).

**Fig. 3:**
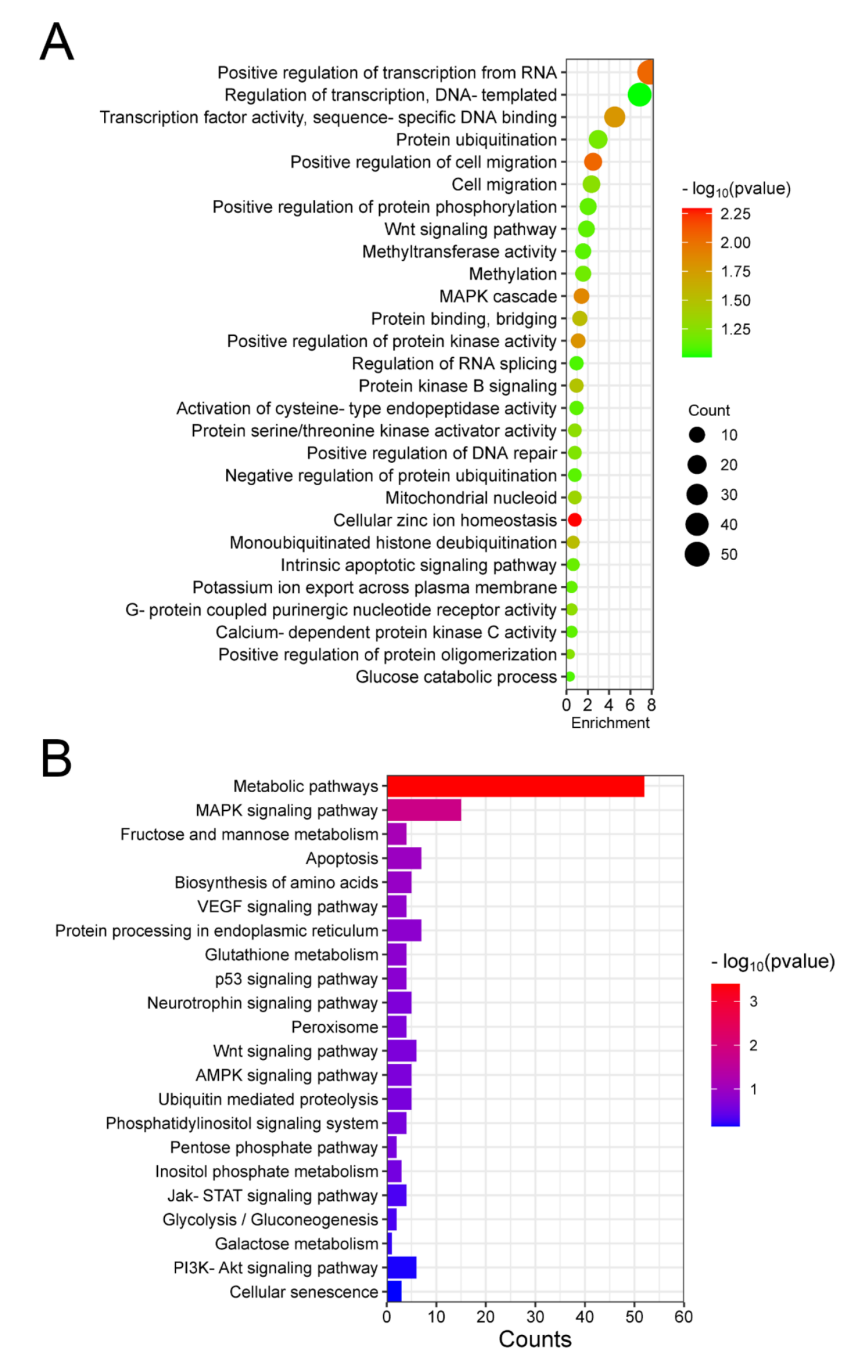
Gene Ontology (GO) enrichment and KEGG pathway analysis was performed for significantly upregulated DEGs. (**A**) GO identifiers in the cluster of significant upregulated DEGs in resistant mice compared to sensitive mice. (**B**) KEGG pathway analyses of upregulated DEGs in resistant mice compared to sensitive mice.

To determine the functions of the identified differentially expressed genes (DEGs), a Gene Ontology (GO) enrichment analysis was performed for significantly upregulated DEGs (Fig. 3A). The GO results showed that upregulated DEGs were involved in protein modifications and protein kinase activity regulation. Specifically, protein modifications include: “Protein binding, bridging”, “Monoubiquitinated histone deubiquitination”, “Methylation”, “Protein ubiquitination” and “Positive regulation of protein phosphorylation”, and protein kinase activity regulation includes: “Protein kinase B signaling”, “Protein serine/threonine kinase activator activity”, “Positive regulation of protein kinase activity”, “G-protein coupled purinergic nucleotide receptor activity”, “Calcium-dependent protein kinase C activity” and “Methyltransferase activity”. To further determine the specific signaling pathway, the significantly upregulated DEGs were analyzed using the KEGG pathway database (Fig. 3B). The significantly upregulated DEGs were observed to be mainly including: “Metabolic pathways”, “MAPK signaling pathway”, “AMPK signaling pathway”, “PI3K-Akt signaling pathway”, “Jak-STAT signaling pathway”, “p53 signaling pathway” and “Wnt signaling pathway”. These results indicate that the protein modifications and metabolism-related physiological functions of cells in the PrL of resistant mice were activated, and these protein modifications could regulate the structure, stability, function, and interactions of proteins, thereby affecting intracellular signal transduction, metabolism, and other biological processes. Although the expression levels of genes involved in these biological processes were significantly increased in resistant mice, we were unable to determine whether this could affect the behavior of resistant mice.

**Fig. 4:**
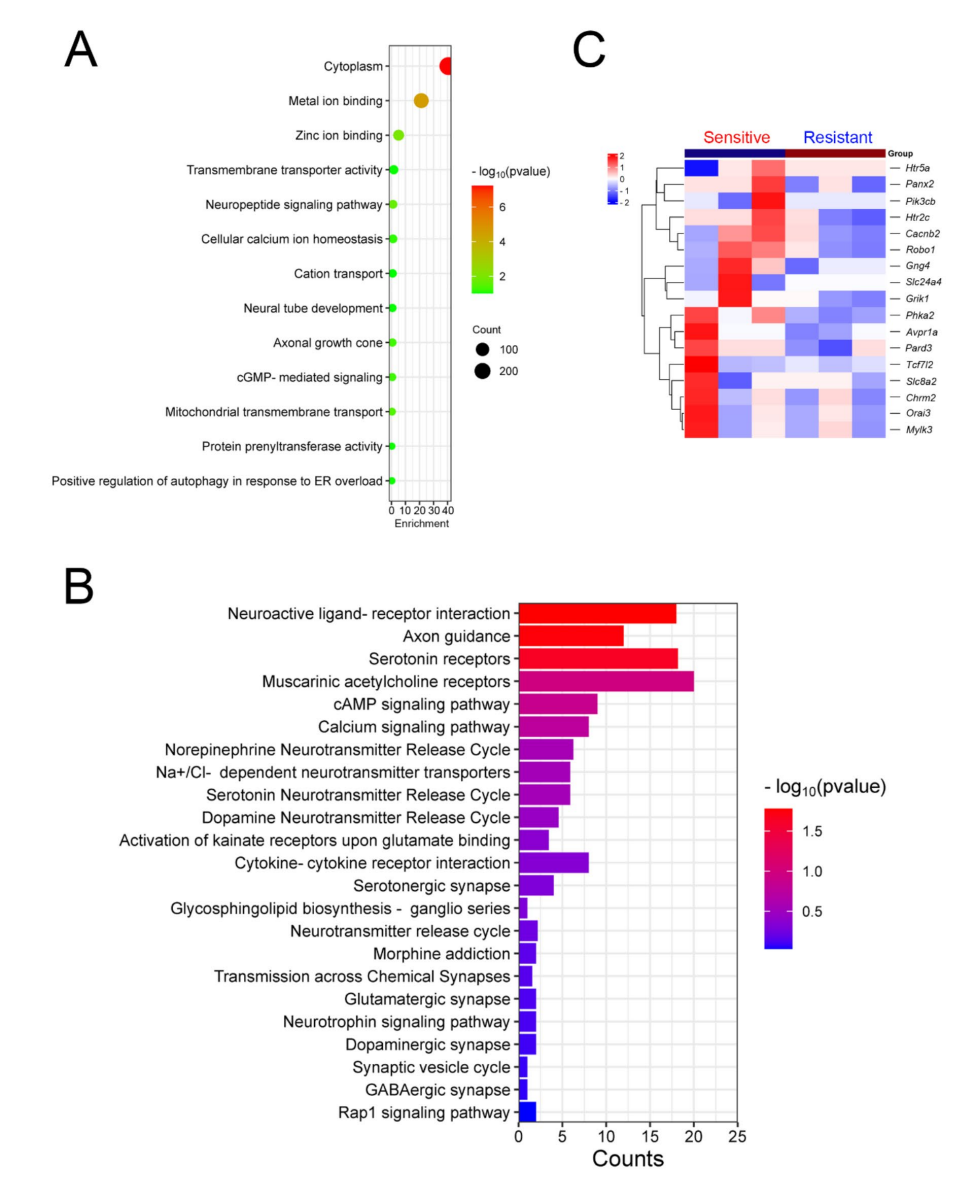
Gene Ontology (GO) enrichment and KEGG pathway analysis was performed for significantly downregulated DEGs. (**A**) GO identifiers in the cluster of significant downregulated DEGs in resistant mice compared to sensitive mice. (**B**) KEGG pathway analyses of downregulated DEGs in resistant mice compared to sensitive mice. (**C**) Heatmap showing the expression of representative DEGs between resistant and sensitive mice.

Furthermore, we also performed GO and KEGG enrichment analyses for significantly downregulated DEGs. The GO results showed that downregulated DEGs were involved in ion homeostasis and neuronal development (Fig. 4A). Such as: “Cellular calcium ion homeostasis”, “Cation transport”, “Zinc ion binding”, “Neural tube development”, “Axonal growth cone” and “Neuropeptide signaling pathway”. Additionally, the KEGG results showed that downregulated DEGs were involved in synapse types, neuronal signal transduction and neurotransmitter release (Fig. 4B). Specifically, synapse types including: “Serotonergic synapse”, “Glutamatergic synapse”, “Dopaminergic synapse”, “GABAergic synapse”, “Serotonin receptors” and “Muscarinic acetylcholine receptors”. The neuronal signal transduction including: “Neuroactive ligand-receptor interaction”, “cAMP signaling pathway”, “Calcium signaling pathway”, “Neurotrophin signaling pathway”, and “Na^+^/Cl^−^ dependent neurotransmitter transporters”. The neurotransmitter release including: “Synaptic vesicle cycle”, “Norepinephrine neurotransmitter release cycle”, “Serotonin neurotransmitter release cycle” and “Dopamine neurotransmitter release cycle”. These results indicate that genes involved in regulating neural system functions were downregulated in resistant mice (Fig. 4C), which may lead to a decrease in synaptic types, impairment in neurotransmitter release, and damage to the information exchange functions between neurons. Therefore, we speculated that there may be defects in the neuronal system of resistant mice, and the decreased expression levels of genes involved in regulating neural system functions were the fundamental reasons for the differences between resistant and sensitive mice.

## Discussion

This study aims to investigate the underlying causes of addiction-related behaviors. Using a CPP model with footshock, we successfully simulated morphine-induced CPP, and effectively differentiated between mice resistant (approximately 24%) and sensitive (approximately 76%) to punishment. The proportion of mice displaying addiction-related behaviors is consistent with previous findings^11,30^. Additionally, previous studies have reported neuronal plastic changes in the prefrontal cortex neurons caused by long-term drug use^11^, and there is extensive research on individual differences and molecular changes in addiction models^38,39^. In the present work, we found that regulating neuronal activity in the PrL could modulate morphine-induced CPP behavior. Furthermore, transcriptomic sequencing analysis revealed potential neuronal functional defects in the PrL of resistant mice.

In humans, some individuals could achieve self-control after drug use through certain withdrawal methods, but a small fraction of people, even after withdrawal, still cannot control themselves and exhibit compulsive addictive behavior^40,41^. However, it is difficult to predict who will develop addiction and who will not. Animal studies could provide valuable information about the factors that may increase the risks of developing addiction. We found that a minority of mice still exhibit morphine-induced CPP behavior even after experiencing footshock. However, the reasons behind addiction-related behavior in resistant mice are still uncertain. Some studies suggest that addiction-related behavior is associated with uncontrollable habits^42,43^. For example, Jones BO, et al.^44^, found that punishment resistant was related to habits that have become inflexible and persist under conditions that should encourage a transition to goal-directed behavior. Leong KC, et al.^45^ suggested that the full acquisition and relapse of addiction-related behavior may be attributed to a shift away from goal-directed responding and a shift towards the maladaptive formation of rigid and habit-like responses. This habitual response can be acquired through long-term training, characterized by insensitivity to changes in outcomes. The morphine-induced CPP behavior observed in resistant mice in our study showed habitual preference, and the decreased flexibility between habitual and the goal-directed systems are key factors in compulsive drug use^46,47^.

Additionally, PFC play a broader role in mediating adaptive responding and behavioral flexibility^47^, some studies indicating that defects in cortical neuron function and projections involving different neural circuits contribute to addiction-related behavior. For example, Chen Y, et al.^30^ found that anterior insular cortex (aIC) glutamatergic neurons and the OFC-aIC circuit gated the shift from controlled to compulsive cocaine use. Wang L, et al.^48^ found that frontal association cortex (FrA) as a critical region mediating cocaine-associated locomotor sensitization, and the dopamine neurons activity decreased in FrA is one of the essential mechanisms underlying cocaine-induced place preference and compulsive seeking behaviors. Chen BT, et al.^11^ suggest that prolonged cocaine use depressed prelimbic cortex excitability, and that, in a select population of rats, a profound prelimbic cortex hypoactivity drove compulsive cocaine seeking. Consistent with these findings, we found that activating of excitatory neurons in the PrL region of resistant mice effectively suppresses their morphine-induced CPP behavior. Conversely, inhibition pyramidal neurons in the PrL of sensitive mice induces morphine-induced CPP behavior. These findings underscore the pivotal role of PrL neuronal activity in the addiction process.

Although various factors including habitual behavior, goal-directed behavior, neural projections, etc., lead to drug seeking and compulsive addictive behaviors, these factors may differ across individuals (i.e., individual differences). Therefore, we explored individual differences between resistant and sensitive mice by RNA-seq analysis. We observed a large number of genes related to the growth and development of nervous system and neuronal function were significantly down-regulated in PrL of resistant mice compared with sensitive mice. Specifically, *Pard3*, *Robo1*, *Panx2*, and *Tcf7l2* are mainly involved in the regulation of neuronal development and migration; *Htr2c*, *Grik1*, *Chrm2*, *Avpr1a* and *Htr5a* are primarily involved in the regulation of synaptic receptor formation; *Orai3*, *Phka2*, *Slc8a2*, *Slc24a4* and *Cacnb2* primarily affect neuronal activity and function by regulating calcium ion activity.

The development of the mammalian cerebral cortex not only affects neurological disorders but may also trigger various mental illnesses, such as epilepsy, autism and bipolar disorder^49^. *Pard3*, par-3 family cell polarity regulator, involved in regulating cortical development and neuronal migration^50^. Pard3 dysfunction drives cortical enlargement with massive heterotopia^51^. *Robo1*, roundabout guidance receptor 1, encodes an integral membrane protein that functions in axon guidance and neuronal precursor cell migration, as well as regulating axon trajectories and suppressing formation of ectopic axons^52^. *Panx2*, Pannexin-2, is involved in many processes of neuronal development, including neurite outgrowth, dendritic spine formation, and N-methyl-D-aspartate (NMDA) receptor (NMDAR)-dependent plasticity^53^. Additionally, the absence of *Panx2* lead to neuronal hyperexcitability and induce neurologic disorders such as epilepsy^54^. *Tcf7l2*, TCF4 transcription factor 4 play an important role in nervous system development, and *Tcf7l2* loss of function alters the intrinsic excitability of prefrontal neurons^55^. *Pard3*, *Robo1*, *Panx2*, and *Tcf7l2* were significantly decreased expression in the PrL of resistant mice, suggesting potential defects in cortical neurodevelopment in resistant mice.

Furthermore, brain development encompasses a number of processes including synaptogenesis and synaptic plasticity^56^, and neurotransmitter receptors play crucial roles in neurotransmission and neuronal function regulation. We also detected significantly decreased expression levels of genes involved in synaptic receptor formation in the PrL of resistant mice, including *Htr2c*, *Htr5a*,* Grik1*,* Avpr1a* and *Chrm2*, which may likely impact the functioning of the nervous system in resistant mice. *Htr2c* and *Htr5a* encode a seven-transmembrane G-protein-coupled receptor, 5-hydroxytryptamine receptor, involved in impaired executive function in a wide range of psychiatric conditions^57^. The decrease of *Htr2c* expression level leads to the defected of neuronal activity^58^, and lack of *Htr5c* lead to bipolar disorder and schizophrenia^57,59^. *Grik1*, glutamate ionotropic receptor kainate type subunit 1, encode glutamate receptors, which is the major excitatory neurotransmitter receptor in the mammalian brain. Reduced expression of *Grik1* lead to impaired excitatory neurotransmission and exacerbated inhibitory GABAergic transmission, which could trigger psychiatric disorders such as anxiety and epilepsy^60^.

Moreover, the functioning of Na/Ca exchanger molecule plays a critical role in calcium homeostasis in neurons, ultimately impacting neuronal activity^61^. *Orai3*, ORAI calcium release-activated calcium modulator 3, involved in store-operated calcium entry^62^. Loss of *Orai3* will lead to reduced neuronal excitability and affect synaptic plasticity^63^. *Slc8a2*, solute carrier family 8 member A2, involved in regulation of postsynaptic cytosolic calcium ion concentration and calcium/sodium antiporter activity^64^. Decreased expression levels of *Slc8a2* will affect synaptic vesicle release and impact learning and memory^65^. *Slc24a4*, solute carrier family 24 member, also known as *Nckx4*, encodes a sodium/potassium/calcium exchange protein^66^. And plays as a powerful extrusion pathway that assists in terminating Ca^2+^ signals^67^. Knocking down *Slc24a4* increases the severity of ischemic brain injury and leads to neuronal damage. *Cacnb2*, calcium voltage-gated channel auxiliary subunit beta 2, encodes a subunit of a voltage-dependent calcium channel protein that is a member of the voltage-gated calcium channel superfamily^68^. Knocking down *Cacnb2* lead to imbalance in neuronal calcium homeostasis and dendritic impairments, severe cases of which could induce neurocognitive dysfunction and Bipolar disorder^69^. We found a significant downregulation of the above-mentioned genes involved in calcium ion homeostasis regulation in the PrL of resistant mice, which may lead to neuronal activity disruption and affect addiction-related behavior.

In conclusion, this study found that individual differences among mice could affect addictive behaviors, with resistant mice were more prone to morphine-induced CPP behavior compared to sensitive mice. Activating the activity of PrL pyramidal neurons could alter morphine-induced CPP behavior in resistant mice, indicating that the activity of prefrontal cortex neurons in resistant mice may be relatively lower. Additionally, we found a significant downregulation of gene expression affecting neuronal activity in the PrL of resistant mice, suggesting that this may be the underlying cause of individual differences between resistant and sensitive mice. However, this study still has limitations. For example, there is a lack of assessment of the potential effects of CNO on mouse behavior, and the concentration of CNO used is relatively high. Although literature has reported that different concentrations of CNO (0.1, 0.3, 1, 3 mg/kg, i.p.) do not affect mice’s reward-seeking behavior or operant learning behavior^70,71^, we will pay attention to this issue in future research. Additionally, this study used a gradient morphine administration to simulate the real process of drug addiction, which may lead to withdrawal behaviors in the mice; we will also consider this issue in future studies. Furthermore, we found that a small number of sensitive mice (3 out of 37) exhibited morphine-induced place preference in Test 3 (Fig. 1C), which may be due to drug-associated memory overcoming punishment-related memory, and this requires further in-depth investigation. Although further research is needed to elucidate the role of these genes in addition, our findings may contribute to understanding addiction-related behavior and development of related therapeutic approaches for drug addiction.

## Materials and methods

### Animals

C57 (BL6/J) mice were purchased from Guangdong Medical Laboratory Animal Center (China). All experiments have been approved by the Peking University Shenzhen Graduate School Animal Care and Use Committee (Permit Number: AP0011) and were performed in accordance with the ARRIVE guidelines on the Care and Use of Experimental Animals. We confirmed that all experiments were performed in accordance with relevant guidelines and regulations. 3–5 months male mice were used. All mice were maintained under the standard laboratory conditions at 22 ± 2 °C, with 50 ± 10% relative humidity, and on a 12 h light/dark cycle, with food and water available ad libitum.

### Conditioned place preference

The CPP was conducted using a biased protocol and based on prior literature with slight alterations^72^. The chambers for testing conditioned place preference (CPP) test were consisted of two distinct Perspex compartments and separated by manual guillotine-style doors. Each compartment has the same size (22 cm width× 22 cm length × 30 cm height) and distinct contextual characteristics, one with a white-striped wall and stainless-steel grid floor (transverse), and the other with a black wall and stainless-steel grid floor (longitudinal). During use, the CPP chambers are illuminated with dim lighting (90 lx). Immediately following use entire preference chamber was cleaned thoroughly with 75% alcohol. Mouse positions, movement trajectories, and time spent in each compartment are automatically analyzed using the automated data collection software (Any-maze software).

The CPP experimental protocol is illustrated in Fig. 1A, and mainly consists of habituation, conditioning, and test phases. Specifically, during the Habituation phase, mice are placed in the compartments once a day for 15 min, with two consecutive days of free access to all compartments. The time spent in each compartment (in seconds) is recorded to evaluate and select mice without location preference and normal motor abilities for the next step of experiments. Next, the mice undergo conditioning training for 8 days (days 3–10), during which the connection between two compartments was closed. To more closely mimic the real pattern of drug addiction, we chose to the protocols of Zhu et al.^73,74^. and used a gradient concentration of morphine for modeling. Morphine injections (i.p.) are administered at gradient concentrations (10, 20, 30, 40 mg/kg), while the control group receiving saline instead of morphine. After injection, the mice were placed in the conditional compartment for 45 min for adaptation. After 24 h, the mice were injected with saline (i.p.) and placed in the opposite compartment for adaptation. They were injected only once per day, with either morphine or saline. After four cycles of this protocol, the adaptation training is concluded. Finally, during the testing phase, the barrier between the two compartments was removed, allowing the mice to move freely between them. The testing duration is 15 min (Test 1), and the CPP score is calculated by subtracting the time spent in the morphine-paired compartment from the time spent in the other compartment. The preference of the mice was assessed according to CPP scores, and punishment training was conducted 24 h later. Whenever the mice entered the compartment paired with morphine, a 2-second electric shock (0.75 mA) is administered as pervious study^75^, and the training duration 30 min. After 24 h, the second test (Test 2) is conducted, using the same testing method as Test 1. After 24 h, the third test (Test 3) is conducted to determine evaluate the stability of the experiment, using the same testing protocol as Test 1. Mice were categorized into resistant and sensitive groups based on their CPP scores.

### Virus injection

As described in previous study^48^, mice were anesthetized with isoflurane (induction 3%, maintenance 1.5%) and then secured onto a digital stereotaxic instrument (RWD Life Science Co., China) for virus injection. The scalp was incised, and the skull was scrubbed with iodine and rinsed with physiological saline. Unless otherwise stated, all coordinate measurements at different locations were relative to the bregma. AAV2/9-CaMKIIα-hM3Dq-mcherry (PT-0042, BrainVTA, China) or AAV2/9-CaMKIIα-hM4Di-mcherry (PT-0017, BrainVTA, China) was injected into the PrL region (AP + 2.00 mm, ML ± 0.40 mm, DV -1.28 mm) respectively. The injection volume was 200 nL, controlled at a speed of 40–50 nl/min. After injection, the needle was left in place for approximately 5 min, then physiological saline was used to seal the needle hole, followed by slow withdrawal of the needle. After suturing the incision, the mice were transferred to a heating pad until they regained consciousness. Clozapine-N-oxide (CNO) was dissolved in dimethylsulfoxide (DMSO) in 0.9% saline, and administered intraperitoneally (i. p.) at a dose of 3.0 mg/kg as previous studies, and the dosage was chosen based on prior work showing that 3 mg/kg is an effective dose^47,76^. The behavioral assays were performed about 30 min after the injection of CNO.

### RNA-seq and differential gene expression analysis

The materials used for RNA-seq assay were obtained from the PrL region of mouse brain, with three samples per group. The RNA-seq sequencing work was primarily carried out by Biomarker Technologies Co. Ltd. Briefly, the TruSeq RNA sample preparation kit (Illumina, San Diego, CA, USA) was used for RNA-seq library preparation^48^. Then, sequencing of the libraries was conducted on an Illumina HiSeqTM 3000 system. A computational pipeline was used to process RNA-seq data. Sequence data were mapped to the mouse reference genome GRCm38 with Tophat (v2.0.12) with default parameters. And calculated the expression level of genes based on fragments per kilobase of transcript per million fragments mapped (FPKM) using Cufflinks software^77^. Finally, the differentially expressed genes (DEGs) between resistant and sensitive to identify significantly up- or downregulated genes. The DEGs were assessed by DESeq 2 (v1.16.1) software (https://bioconductor.org) with the Benjamini–Hochberg multiple test correction method. The DEGs were selected according to |log2 fold change| ≥ 1 and adjusted p value < 0.05. Gene Ontology (GO) and Kyoto Encyclopedia of Genes and Genomes (KEGG) (http://www.genome.jp) annotations for enrichment^78^. The heatmaps were plotted by Bioinformatics (https://www.bioinformatics.com.cn).

### Data analysis

Statistical analyses were performed with GraphPad Prism 9. A one-way repeated measure ANOVA was conducted to compare the measured cpp score at conditions before and after of footshocks. Bonferroni test was used for post hoc analysis after ANOVA. Data were presented as mean ± SEM. Significance was defined as *P < 0.05, **P < 0.01.

## Electronic supplementary material

Below is the link to the electronic supplementary material.

Supplementary Material 1

## Data Availability

All data analysed during this study are included in this published article and its supplementary information files.
